# Caregiver‐oriented needs and priorities in socio‐cognitive assessment of neurocognitive disorders: a multi‐stakeholder pilot study from the *SIGNATURE* network

**DOI:** 10.1002/alz70857_104313

**Published:** 2025-12-25

**Authors:** Andrea Panzavolta, Alessandra Dodich, Giulia Funghi, Claudia Meli, Renzo Dori, Helena Briales, Laura Invernizzi, Mario Possenti, Jordi A Matias‐Guiu, Chiara Cerami

**Affiliations:** ^1^ Scuola Universitaria Superiore IUSS, Pavia, Pavia, Italy; ^2^ University of Trento, Rovereto, Trento, Italy; ^3^ Associazione Alzheimer Trento ODV, Trento, Trento, Italy; ^4^ Asociación Ayuda Afasia, Madrid, Madrid, Spain; ^5^ Associazione Italiana Malattia Frontotemporale, AIMFT, Brescia, Brescia, Italy; ^6^ Federazione Alzheimer Italia, Milan, Milan, Italy; ^7^ Hospital Clinico Universitario San Carlos, Madrid, Madrid, Spain; ^8^ ICS Maugeri IRCCS, Milan, Milan, Italy

## Abstract

**Background:**

Socially inappropriate behaviors and changes in the social cognition domain characterize the clinical phenotype of different neurocognitive disorders (NCDs) and have a crucial impact on the patient‐caregiver dyad's quality of life. Notably, there is a lack of tailored interventions targeting these impairments and various outcome measures have been used in the few available studies in the field, limiting comparability.

**Objective:**

This work describes the preliminary activities for a two‐stage project within Phase 3 of the international *SIGNATURE* initiative aimed at identifying a comprehensive set of patient‐oriented concepts of interest that are meaningful for socio‐cognitive clinical research in NCDs.

**Materials & Methods:**

A self‐administered online survey was distributed through the international *SIGNATURE* network (https://sites.google.com/unitn.it/signature‐initiative) to patient and caregiver associations. It included general information on the respondent, *ad hoc* developed scales to explore the perceived clarity and utility of neuropsychological assessment, perceived relevance of socio‐cognitive testing, and priorities for the assessment of social behavior changes in clinics.

**Results:**

Six Italian and Spanish caregiver associations answered the call. The respondents were 198 (69% females; 57±12 years of age; 15±3 years of education). 74% of responders agree that assessment is useful in understanding the course of disease and 61% in understanding deficits in daily life. However, more than 50% of the sample reported crucial difficulties in comprehending scoring table and final commentary, emphasizing the need for dedicated legends on scores and for non‐specialist terminology. While testing of socio‐cognitive abilities was deemed equally relevant (80%) to other cognitive domains, measuring real‐life social behaviors such as empathic response, social aggregation, and socio‐emotional regulation was considered as crucial.

**Conclusion:**

This pilot study underscores the need of promoting educational initiatives for both clinicians and stakeholders to enhance the clarity of socio‐cognitive assessment for the benefit of patients and caregivers. Measures of real‐life social behavior changes should be crucially developed and tested as core outcomes in socio‐cognitive clinical research.

